# Polysaccharides from Basidiocarps of the Polypore Fungus *Ganoderma resinaceum*: Isolation and Structure

**DOI:** 10.3390/polym14020255

**Published:** 2022-01-08

**Authors:** Roman Bleha, Lucie Třešnáková, Leonid Sushytskyi, Peter Capek, Jana Čopíková, Pavel Klouček, Ivan Jablonský, Andriy Synytsya

**Affiliations:** 1 Department of Carbohydrates and Cereals, UCT Prague, 166 28 Prague, Czech Republic; Lucie.tresnakova@gmail.com (L.T.); Sushytskyi@vscht.cz (L.S.); copikovj@vscht.cz (J.Č.); 2 Institute of Chemistry, Slovak Academy of Sciences, Dúbravská cesta 9, 842 38 Bratislava, Slovakia; Peter.Capek@savba.sk; 3 Department of Gardening, Faculty of Agrobiology, Food and Natural Resources, Czech University of Life Sciences Prague, 165 00 Prague, Czech Republic; kloucek@af.czu.cz; 4 Department of Crop Production, Faculty of Agrobiology, Food and Natural Resources, Czech University of Life Sciences Prague, 165 00 Prague, Czech Republic; i.jablonsky@seznam.cz

**Keywords:** *Ganoderma resinaceum*, wood decay fungi, polysaccharides, spectroscopy

## Abstract

In this study, we focused on the isolation and structural characterization of polysaccharides from a basidiocarp of polypore fungus *Ganoderma resinaceum*. Polysaccharide fractions were obtained by successive extractions with cold water at room temperature (20 °C), hot water under reflux (100 °C), and a solution of 1 mol L^−1^ sodium hydroxide. The purity of all fractions was controlled mainly by Fourier transform infrared (FTIR) spectroscopy, and their composition and structure were characterized by organic elemental analysis; neutral sugar and methylation analyses by gas chromatography equipped with flame ionization detector (GC/FID) and mass spectrometry detector (GC/MS), respectively; and by correlation nuclear magnetic resonance (NMR) spectroscopy. The aqueous extracts contained two main polysaccharides identified as a branched *O*-2-*β*-d-mannosyl-(1→6)-*α*-d-galactan and a highly branched (1→3)(1→4)(1→6)-*β*-d-glucan. Mannogalactan predominated in the cold water extract, and *β*-d-glucan was the main product of the hot water extract. The hot water soluble fraction was further separated by preparative anion exchange chromatography into three sub-fractions; two of them were identified as branched *β*-d-glucans with a structure similar to the corresponding polysaccharide of the original fraction. The alkaline extract contained a linear (1→3)-*α*-d-glucan and a weakly branched (1→3)-*β*-d-glucan having terminal *β*-d-glucosyl residues attached to *O*-6 of the backbone. The insoluble part after all extractions was identified as a polysaccharide complex containing chitin and *β*-d-glucans.

## 1. Introduction

Wood decay polypore fungi of the genus *Ganoderma*, some species of which have long been cultivated used in traditional oriental medicine [1,2,3,4], are widespread in warm regions and are often found in parks and gardens [5]. These fungi have thick, corky fruiting bodies (basidiocarps), which are capable of growing on affected dead or living trees at the site of injury and causing white rot. They decompose lignocellulosic complex of the wood using a number of lignin-modified enzymes, including laccases, lignin peroxidase, and manganese-dependent peroxidase [6,7]. As a result, the wood loses its hardness, strength, and mass. The destruction of lignin leads to a lightening of the color of the wood [8,9].

Fungi of genus *Ganoderma* produce secondary metabolites such as proteins, polysaccharides [10,11,12,13,14,15,16,17,18], terpenoids [14,16,17,19,20], polyphenols [21,22], steroids, alkaloids, and many others with various biological effects [1,23,24,25,26]. Due to these properties, metabolites of *Ganoderma* are used in functional foods, food supplements, or cosmetics [27,28,29].

Unlike the low molecular weight metabolites mentioned above, in higher fungi including *Ganoderma*, most polysaccharides, as well as some proteins and glycoproteins, primarily play the role of structural components of the cell walls of spores, mycelium, and fruiting bodies, where they are interconnected and form a layered three-dimensional basis [30,31,32]. To isolate individual polysaccharides from the fungal material, it is necessary to break numerous durable bonds between them. For this purpose, extraction with various solvents and reagents under appropriate conditions is usually used [33]. Before proceeding with the isolation of polysaccharides, the crushed basidiocarp is first washed with organic solvents to remove lipids and other low molecular weight substances [18]. Hot water extraction is commonly used for obtaining polysaccharides from basidiocarp and other parts of mushrooms *Ganoderma* [34,35]. This method does not require any special reagents and makes it possible to obtain a water-soluble polysaccharide fraction most suitable for use. Heating up to the boiling point usually allows the separation of heteropolysaccharides and some glucans from the cell walls of fungi, often mixed with proteins and glycoproteins. In some cases, however, extractions are used first with cold and then with hot water to separate the individual water-soluble fractions [36]. Acidic, saline, and alkaline solutions are also used for the extraction of fungal polysaccharides [37,38]. Here, multiple extractions are often used under moderate to high conditions to gradually destroy the cell walls from the periphery to the inside and thus obtain polysaccharide fractions with different solubility [18]. In addition to the above, recently, several advanced methods, such as hot compressed water, subcritical or two-phase extraction, microwave heating, ultrasound, enzymes, and their combinations have been used to more effectively destroy cell walls and thus increase the yield of fungal polysaccharides [39,40,41,42,43,44,45].

The crude polysaccharide fractions are subjected to further purification steps, including the removal of proteins by hydrolysis with proteolytic enzymes or precipitation using various reagents [46]. At this stage, mild conditions should be preferred to avoid partial degradation of the polysaccharides. The final purification step includes dialysis and preparative chromatography, usually a combination of anion exchange and gel filtration methods [18]. Then, the purity, composition, molecular weight, and structure of the obtained polysaccharides are analyzed by appropriate spectroscopic and separation methods [33,47,48].

In general, linear and branched d-glucans of *α*- and *β*- and mixed *α*,*β*-anomeric configurations are very common in fungi including *Ganoderma*, while cellulose or (1→4)-*β*-d-glucan are not found in these organisms [30]. Some heteropolysaccharides of various structures and composition are also found in *Ganoderma*, but mainly in the water-soluble part [49,50,51]. These include, for example, heterogalactans [52,53] and heteromannans [54]. In contrast, chitin is present in fungi as rigid component of cell walls in complex with d-glucans [34].

The most studied water and alkali-extracted polysaccharides have been isolated from *Ganoderma lucidum,* also known as Linzhi or Reishi, which has been used for centuries in Asian medicine [11,14,16,55,56,57]. These polysaccharides were shown to be mainly glucans of various structures, including (1→3)(1→6)-*β*-d-glucan, *β*-(1→3)-glucan, and *α*-(1→4)-glucan. The minor structural monosaccharides were found to be xylose, arabinose, galactose, mannose, and fucose. Various polysaccharides of *Ganoderma* have positive health effects such as immunomodulatory [44,58,59], antimicrobial [14,15], antiviral [60,61], prebiotic [62], hypoglycemic [63,64,65,66], antioxidant [14,15], anti-inflammatory [51,67], and antitumor [15,17,51,67] effects. Biological activities of fungal polysaccharides are closely related to the sugar composition, branching, and molecular weight, which in turn are closely related to the isolation procedure [47,68].

On the other hand, the polysaccharides of other species of the genus *Ganoderma* have been insufficiently studied. Moreover, species such as *G. resinaceum* may be interesting as sources of various biologically active polysaccharides, structurally different from the polysaccharides of *G. lucidum*, since certain species of this genus show a certain specificity in biochemical composition [59,69]. In this regard, it may be promising to grow some unconventional species of the genus *Ganoderma* to obtain not only polysaccharides, but also other biologically active substances, such as terpenoids or polyphenols. Partially, the mentioned species *G. resinaceum* is quite widespread in Central Europe, common in parks and forests, and is currently considered promising for experimental cultivation.

Therefore, this work is devoted to the isolation, structural analysis, and composition of polysaccharide-rich fractions obtained from *Ganoderma resinaceum* basidiocarp*,* originated from the Czech Republic. These fractions were purified by chemical and enzymatic treatments enzymes, and further separated by ion exchange chromatography. Composition and structure of obtained polysaccharides were characterized by spectroscopic, chromatographic, and other analytical methods.

## 2. Materials and Methods

### 2.1. Materials

**Figure 1:**
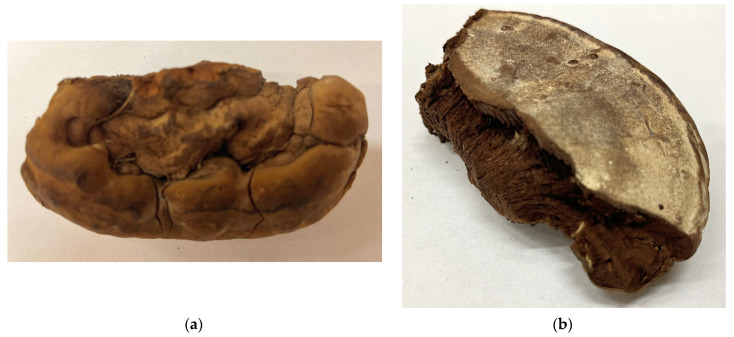
Part of basidiocarp of *G. resinaceum* used in this work: (**a**) top view; (**b**) bottom view.

Dried and homogenized basidiocarp from the fungus *Ganoderma resinaceum* was found in the town park in Kralupy nad Vltavou, Middle Bohemian region, Czech Republic. This basidiocarp (Figure 1a,b) was identified by Dr. Michal Tomšovský (Mendel University in Brno, Czech Republic), who is a specialist in mycology of forest wood decay fungi, and the specimen was deposited in the Herbarium of Moravian Museum (BRNM), Brno, Czech Republic. This raw material was dried in air and homogenized to a particle size of 0.5 mm using the IKA homogenizer (IKA Werke, Staufen, Germany).

Chemicals:

- Distilled water, ethanol (University of Chemistry and Technology Prague, Czech Republic).
- Methanol, hexane, acetone, dichloromethane, acetanhydride, hydrochloric acid, sulfuric acid, sodium chloride, sodium hydroxide, hydrogen peroxide (PENTA s.r.o., Prague, Czech Republic).
- Sodium borohydride, copper(II) chloride, pepsin from porcine gastric mucosa, formic acid, trifluoroacetic acid, *m*-hydroxybiphenyl, methyl iodide, 1-methylimidazole, 2-deoxy-d-glucose (Sigma-Aldrich, Saint Louis, MO, USA).
- Pronase from *Streptomyces griseus* (Roche Holding AG, Basel, Switzerland).
- Dimethyl sulfoxide (ThermoFisher Scientific, Waltham, MA, USA).
- Potassium bromide for IR spectroscopy (Merck, KGaA, Darmstadt, Germany).

### 2.2. Preparative Procedures

**Figure 2:**
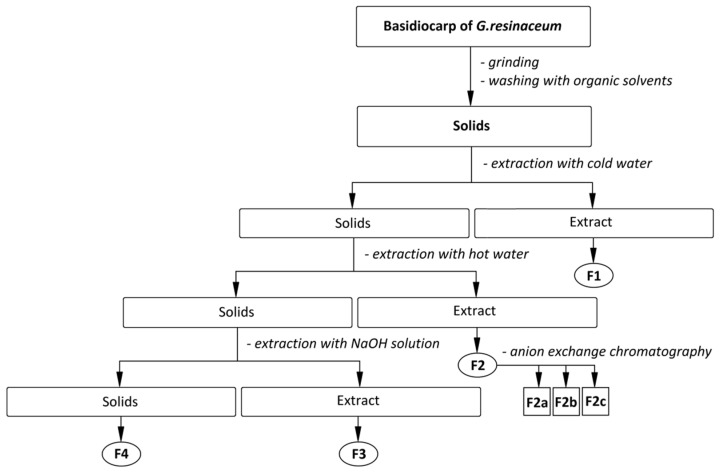
The scheme of isolation of the polysaccharidic fractions **F1**, **F2**, **F3**, and **F4** and sub-fractions **F2a–c** from basidiocarps of *G. resinaceum*.

Before the extraction of polysaccharides, according to the isolation scheme (Figure 2), lipids, pigments, and other small molecules were removed from the homogenized raw material by subsequent washing of the milled material with hexane followed by 0.2 mol L^−1^ HCl in 80% aqueous ethanol (acidified ethanol), 80% aqueous ethanol until neutral reaction, and finally, by 96% ethanol and acetone to remove water residues. Solids after washing with an acidic ethanol mixture were re-homogenized, subjected to recurrent extraction with cold water (20 °C), followed by hot water under reflux (100 °C), and then with a solution of 1 mol L^−1^ aqueous NaOH [68].

Extraction with cold water was performed four times; 50 g of homogenized dry solids and 1500 mL of distilled water were used. The extraction was carried out on a magnetic stirrer at 350 rpm, and the average stirring time was 6 h. The solids were then separated, filtered, and used for another extraction cycle under the same conditions. The extracts were filtrated, combined, and evaporated in a RVO 400 vacuum evaporator (Ingos s.r.o, Prague, Czech Republic) at 65 °C and 40 mBar. The concentrated extract was freeze-dried on Alpha 1–4 LSC lyophilizer (Pragolab s.r.o., Prague, Czech Republic) yielding fraction **F1**, which was further successively washed in the same way as the raw material and dried in air. Further purification was performed using the combination of proteolytic enzymes (pepsin and pronase) to remove proteins. The sample dissolved in 100 mL of 0.01 mol L^−1^ aq. HCl was incubated with pepsin (100 mg) for 12 h and then the enzyme was inactivated by heating to 90 °C for 30 min. The product was then precipitated in an excess of 96% ethanol (3:1 *v/v*); successively washed with 80% ethanol, 96% ethanol, and acetone; dried in air; and homogenized. 

The insoluble part after extractions with cold water was used for further extractions with hot water under reflux (100 °C). The extraction was performed in three portions five times in distilled water (1000 mL each portion) for approximately 8 h. The extracts were used for obtaining of purified fraction **F2** by the same way as described above for **F1**; further purification was performed using anion exchange preparative chromatography [36,70]. Chromatographic fractionation of **F2** (66.67 mg) led to obtaining of three sub-fractions assigned as **F2a**, **F2b,** and **F2c**.

The insoluble part after extractions with hot water was used for two successive extractions in 1 mol L^−1^ aqueous NaOH (700 mL) at 4 °C. The extract was further precipitated in three volumes of 96% ethanol and stored at 4 °C for 12 h. The precipitate was centrifuged and neutralized by washing with the acidic ethanol, then successively washed with 80% ethanol, 96% ethanol, and acetone, and dried in air. The dark-colored precipitate was treated with of 3% H_2_O_2_ in weak alkaline solution (pH ~ 8) to remove colorant residues, and then precipitated with three volumes of 96% ethanol. The precipitate was again neutralized, washed, and dried in the same manner, and finally homogenized in an oscillating mill MM 301 (Retsch GmbH, Haan, Germany) yielding fraction **F3**. The remaining insoluble part after all of the extractions (**F4**) was prepared by the same way as **F3**, but the treatment with 3% H_2_O_2_ took place several times in suspension because of the insolubility of **F4** under alkaline conditions.

### 2.3. Preparative Chromatography

The fraction **F2** (66.67 mg mL^−1^) was used for further fractionation by preparative anion exchange chromatography performed on an Omnifit column, 44 cm high and 1.5 cm in diameter, packed with DEAE-Sepharose Fast-Flow gel (GE Healthcare, Chicago, IL, USA). The flow rate was 1 mL/min. The gradient was 0–2 mol L^−1^ aq. NaCl. The sample was dissolved in 1 mL of distilled water. The eluted fractions were collected using a Gilson FC 203B fraction collector (Gilson Inc., Middleton, WI, USA) with the required number of tubes and with an Ecom DG 3014 vacuum degasser (ECOM spol. s.r.o., Chrastany u Prahy, Czech Republic) driven by a Gilson MP3 pump (Gilson Inc., Middleton, WI, USA). One hundred and ten tubes were collected, and the amount of total carbohydrates in each was estimated according to the phenol sulfuric acid assay (PSA).

### 2.4. Analytical Methods

#### 2.4.1. Phenol-Sulfuric Acid Assay

The phenol-sulfuric acid assay is a rapid colorimetric reaction for the determination of total carbohydrates in samples. PSA was used after preparative chromatography. In 200 μL of each sample (tube—preparative chromatography), 5% phenol solution and 1 mL of 96% sulfuric acid were added. Each tube was thoroughly shaken on an IKA Vortex 2 tube shaker (IKA Werke, Staufen, Germany) tube shaker and then left to stand for 20 min at laboratory temperature. Then, 250 μL of each sample (tube) was transferred to a micro-titer plate and the absorbance was measured at 490 nm on an Epoch TM 2 microplate spectrophotometer (BioTek Instruments, Winooski, VT, USA). The presence of carbohydrates in the sample was then demonstrated by the yellow gold color of the reaction mixture [71]. Chromatograms, calibration curve, and calculations were processed using Excel software (Microsoft, Redmond, WA, USA).

#### 2.4.2. Organic Elemental Analysis

Organic elemental analysis of C, H, N, and S was performed on an Elementar Vario EL III instrument (Elementar Analysensysteme GmbH, Langenselbold, Germany). The manufacturer determined the precision of the method for the simultaneous analysis of 5 mg of the 4-aminobenzene sulfonic acid standard in the CHNS module at <0.1% abs. for each element. The results of the analysis include all combustible sulfur, both organic and inorganic (S^2−^, SO_4_^2−^), as well as all combustible carbon, organically bound and inorganically bound (CO_3_^2−^). The hydrogen content found is affected by the moisture of the supplied sample.

#### 2.4.3. Monosaccharide Composition and Linkages

Quantitative determination of neutral sugars in all polysaccharide fractions obtained was carried out using their alditol acetates [72] by gas chromatography on a Trace GC Ultra (ThermoFisher Scientific, Waltham, MA, USA) equipped with a Restek RT-2330-NB column (0.32 mm × 105 m), a temperature program of 80 °C (12 min)–160 °C (8 °C min^−1^) – 250 °C (4 °C min^−1^, 25 min at 250 °C) – 265 °C (20 °C min^−1^, 10 min at 265 °C), and a flow rate of helium of 1 mL min^−1^.

The gas chromatograph was coupled to a TSQ Quantum XLS mass spectrometer (GC/MS) (ThermoFisher Scientific, Waltham, MA, USA) with EI ionization 70 eV and mass range of 50 to 650 amu with a scan time of 1 s [73]. Sugar linkage analysis of **F2** was performed using the methods of Ciucanu and Purdie [74,75]. The permethylated products were hydrolyzed with 90% HCOOH at 100 °C for 1 h and then with 2 mol L^−1^ TFA at 120 °C for 1 h, followed by reduction with NaBD_4_ and acetylation. The partially methylated alditol acetates were analyzed by GC/MS.

The content of uronic acids in the hot water extract was measured by photometry with *m*-hydroxybiphenyl at 520 nm [76].

#### 2.4.4. FTIR Spectroscopy

The FTIR spectra of the raw material and all fractions obtained were measured on a Nicolet 6700 FTIR spectrometer in KBr pellets under the given conditions. The wavelength range was 400–4000 cm^−1^, with a resolution of 2 cm^−1^, with 64 scans. Vibration spectra were recorded and processed using Omnic 8.0 software (ThermoFisher Scientific, Waltham, MA, USA). The raw spectra were exported in ASCII format to Origin 6.0 software (OriginLab, Northampton, MA, USA) for further processing (smoothing, baseline correction) and calculation of the average spectra.

#### 2.4.5. FT Raman Spectroscopy

FT Raman spectra of selected fractions (range 100–4000 cm^−1^, 1000 scans, resolution 4 cm^−1^) were recorded on Nicolet iS50 with the FT Raman module (ThermoFisher Scientific, Waltham, MA, USA), Nd:YAG laser (λ_ex_ = 1064 nm, power 500 mW), CaF_2_ beam splitter and InGaAs detector. Vibration spectra were recorded and processed using Omnic 9.0 software (ThermoFisher Scientific, Waltham, MA, USA). The raw spectra were exported in ASCII format to Origin 6.0 software (OriginLab, Northampton, MA, USA) for further processing (smoothing, baseline correction), calculation of the average spectra.

#### 2.4.6. NMR Spectroscopy

The selected fractions were analyzed on a Bruker Avance III omet 500 MHz NMR spectrometer (Bruker, Billerica, MA, USA). The water-soluble fractions (**F1**, **F2**) were dissolved in D_2_O for NMR and measured at 20 °C and 80 °C; alkali soluble fraction (**F3**) was dissolved in Me_2_SO-*d*_6_ and measured at the same temperatures. NMR spectra were processed using MestReNova 10.0 software (Mestrelab Research, Santiago de Compostela, Spain).

## 3. Results and Discussion

### 3.1. Yields of Isolation

The yields of the subsequent isolated fractions are summarized in Table 1. The mass yield of the obtained products of successive extractions gradually increased from 0.37% *w/w* for **F1** and was maximum for the insoluble part **F4** (15.59% *w/w*). As a result, the total yield of the entire product after purification was 28.86% *w/w*, which is less than a third of the initial mass of ground basidiocarp. The extraction yields with hot water (2.33% *w/w*) and alkaline solution (10.57% *w/w*) were slightly higher than that previously reported for *G. lucidum* polysaccharides extracted under optimized conditions, i.e., 1.45% *w/w* and 8.30% *w/w*, respectively [34,38].

### 3.2. Organic Elemental Composition

Amounts of organic elements in the fractions obtained from *G. resinaceum* by successive extractions with cold water, hot water, and 1 mol L^−1^ aqueous NaOH, which were further purified, are summarized in Table 2. Carbon and hydrogen are among the basic skeletal elements of all polysaccharides. The fractions also showed the presence of the nitrogen, maximal for **F1** (2.44% *w/w*), and significantly lower amounts of sulfur; both are derived mainly from proteins. These results are consistent with similar studies [45] that analyzed water extracts of *G. lucidum*. The fractions **F2** and **F3** thus contained less nitrogen and sulfur compared to **F1**, which indicates that most proteins are readily soluble in cold water and removed in this way. In the case of **F4**, the nitrogen is also derived from chitin, which is also a component of *Ganoderma* cell walls [77]. 

### 3.3. Monosaccharide Composition and Linkage

Composition of neutral monosaccharides in the fractions is summarized in Table 3. Among neutral sugars, glucose predominated in all of the fractions, especially in **F3** and **F4**. Glucans of various structure predominate in fungal cell walls [47], so it is not surprising that the glucose content in all fractions, with the exception of **F1**, exceeded 85 mol % and even reached about 92 mol % in the water-insoluble fractions. In contrast, in the fraction **F1,** glucose (35.6 mol %) was found together with comparable amounts of galactose (26.9 mol %) and mannose (26.5 mol %). This fraction also contained the highest percentage of fucose (6.7 mol %) and xylose (4.3 mol %). Since mannose and galactose were found in almost equimolar proportions, these sugars are possibly interconnected in the form of mannogalactan (or galactomannan) polysaccharide in a mixture with d-glucans. A similar monosaccharide composition was achieved with cold water extraction of *Pleurotus ostreatus* basidiocarps [36], where the presence of branched mannogalactan was proved by methylation analysis and correlation NMR. Mannose, fucose, and xylose were found as minor sugars in various proportions (less than 5 mol % each) in all of the other fractions analyzed. Galactose was also found in the fraction **F2**, but at content lower than 2%, and the glucose content prevailed. This means that the dominant polysaccharide of this fraction is d-glucan. This fraction also contained 5.5% *w/w* of uronic acids, as determined by photometry. A similar composition was previously determined for *Pleurotus ostreatus* and *G. lucidum* polysaccharides obtained by microwave-assisted extraction [78]. Aqueous extracts of fungal cell walls usually contain the most labile structural elements of the outer layer, and these are primarily heteropolysaccharides, although some glucans may also be present. However, the water-soluble glucans are preferably released later during the hot water extraction [79], so most of the heteropolysaccharides, mainly mannogalactans, were collected by cold water extraction. Subsequent extractions with alkaline solutions usually lead to the release of mainly glucans of various structures. A similar relationship between fungal glucans and mannogalactans was previously observed for various multistep extraction protocols. As described for the *G. lucidum* basidiocarps [34], successive isolation steps including hot 0.9% NaCl, hot water, and alkaline extractions resulted in an increase in glucose, while the contributions of galactose and mannose were decreased, with branching also declining from high to almost negligible.

Sugar linkage analysis made for the more pronounced water-soluble fraction **F2** revealed about 13 types of sugar derivatives, suggesting a complex chemical structure of the polysaccharides (Table 4). This fraction contained five major types of the fragments in descending order: 1,3-linked, terminal, 1,4-linked, 1,3,6-linked, and 1,6-linked glucosyl units. These fragments indicate the presence of different glucans, possibly a mixture of branched (1→3)(1→6)-*β*-d-glucan and non-branched amylose-like (1→4)-α-d-glucan. The 1,6-linked glucosyl units can form chains of non-branched (1→6)-*β*-d-glucan or be part of side chains in the branched *β*-d-glucan. Fragments of mannose, galactose, fucose, and xylose were also found, but their proportion was too low to judge the inter-connection between them and predict the structure of the corresponding heteropolysaccharides. Obviously, the composition and linkages in polysaccharides obtained from fungi of the genus *Ganoderma* will depend on the type and method of extraction. For example, methylation analysis of polysaccharide isolated from *G. atrum* consisted mainly of 1,3-linked, terminal, 1,3,6-linked, 1,4-linked, and 1,6-linked glucosyl units (from 21% to 12% each), but also contained smaller amounts of galacturonic acid, mannosyl and other glucosyl units [80]. In contrast, two polysaccharides GLC-1 and GLC-2 isolated from *G. lucidum* were defined as galactoglucan and glucan, respectively; both composed of 1,6- and 1,3-linked glucosyl units and GLC-1 also contained 1,6-linked galactosyl residues [81]. In any case, hot water extractable polysaccharides from *Ganoderma* are likely to be highly branched and may contain other carbohydrates besides glucose.

### 3.4. Preparative Chromatography

**Figure 3:**
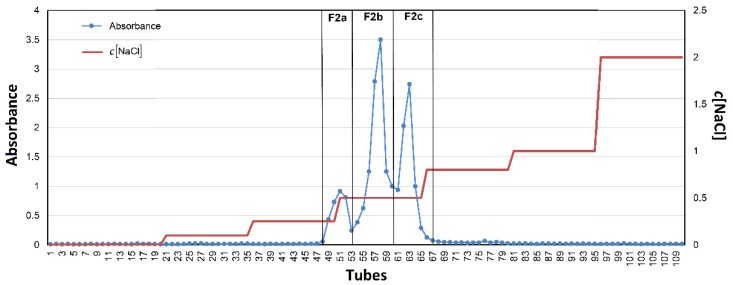
Anion exchange preparative chromatography of **F2**.

A chromatogram describing the fractionation of **F2** is represented in Figure 3. According to the record, the tubes were merged into three sub-fractions, one minor (tubes 47–51) and two major ones (tubes 52–60 and 62–70), assigned as **F2a**, **F2b**, and **F2c**, respectively. All three fractions were dialyzed (1000 Da cut off) and then lyophilized. The yields of these sub-fractions compared to **F2** are summarized in Table 5. 

### 3.5. Vibrational Spectra

**Figure 4:**
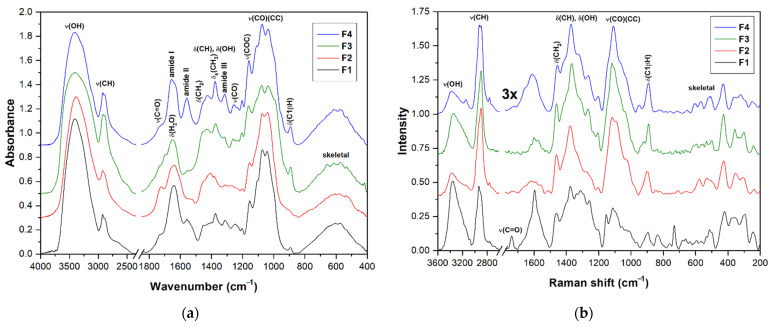
FTIR (**a**) and FT Raman (**b**) spectra of the fractions **F1**, **F2**, **F3**, and **F4** obtained from basidiocarp of *G. resinaceum*.

FTIR and FT Raman spectra of the fractions **F1**–**F4** are shown in Figure 4a,b. The assignment of vibrational bands is given according to the literature [45,48,82,83,84,85]. The spectra of all fractions showed several intense overlapping IR bands in the range of 950–1200 cm^−1^, mainly CC and CO stretching vibrations of the pyranose rings, proving the presence of polysaccharides as the main component of the samples analyzed. An IR band around 1157 cm^−1^ can be assigned to the stretching vibration of the COC glycosidic bonds. Several Raman bands at 1417, 1276, 1205, 1116, 1093, and 1029 cm^−1^ are also characteristic for polysaccharides. A weak IR/Raman band around 897 cm^−1^ from C1H is present in all spectra and is characteristic of *β*-anomeric unit configurations, which could indicate *β*-glucans and, in the case of insoluble part **F4**, also chitin. Additional IR bands and shoulders that can be assigned to *β*-glucans were found near 1375, 1315, 1157, 1073, and 1040 cm^−1^ [84]. The FTIR spectrum of **F4** has three bands at 1656 cm^−1^ (amide I), 1557 cm^−1^ (amide II), and 1317 cm^−1^ (amide III) that indicate the presence of chitin. In the case of the **F1**, the IR bands of amide vibrations around 1648 and 1546 cm^−1^ were assigned to proteins [86]. The presence of chitin and proteins in the mentioned fractions was also confirmed by the significant nitrogen content (see Table 2).

**Figure 5:**
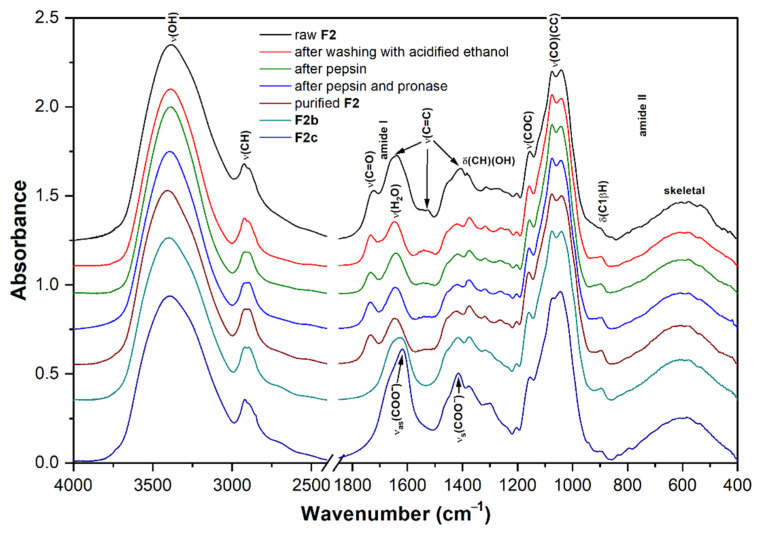
FTIR spectra of the successively purified fraction **F2** and derived sub-fractions **F2b** and **F2c**.

The spectra of the lyophilized raw fraction **F2** and the products of its successive purification including sub-fractions **F2b** and **F2c** obtained by anionic preparative chromatography are shown in Figure 5. In all these spectra, the intense highly overlapped bands at 950–1200 cm^−1^ confirmed the predominance of polysaccharides. Phenolic compounds and other small molecules were removed by washing with 0.2 mol L^−1^ HCl in ethanol, which was confirmed by a decrease in a number of bands related to stretching vibrations of C=C bonds. To remove protein residues, a two-step enzymatic hydrolysis with pepsin and pronase was used. This is because pepsin is capable of cleaving long polypeptide chains into fragments that are still quite large and thus cannot be completely removed. Therefore, for a more complete hydrolysis of proteins, other proteases should be used after pepsin to cleave these fragments into smaller peptides and free amino acids that can be easily removed. The change in the protein content in the fraction can be traced to the intensity of the bands of amide vibrations at about 1647 cm^−1^ (amide I) and 1540 cm^−1^ (amide II) [86]. It should be noted here that the amide I band is not well suitable for the detection of the reminder protein due to overlap with the pronounced scissor vibrations of bound water, and therefore, it is better to use the less intense amide II band for this purpose. As a result, after all purification procedures, the band of amide II became insignificant, which confirms the effective removal of proteins. On the other hand, the two vibration bands of the carboxyl groups at 1740 cm^−1^ (C=O stretching) and 1240 cm^−1^ (CO stretching) remained unchanged after purification, so these groups are most likely part of polysaccharides, possibly in the form of uronic acids [87]. The spectra of sub-fractions did not contain these two bands; instead, the presence of two bands near 1610 cm^−1^ and 1405 cm^−1^, which were assigned to the antisymmetric and symmetric stretching vibrations of the carboxylate anions, respectively, confirmed that the sub-fractions, especially **F2c**, probably contain uronic acids, but in the form of a salt [87]. These two carboxylate bands were not strongly pronounced in the FTIR spectrum of **F2b**; therefore, this sub-fraction contained fewer uronic acids. The spectra of both sub-fractions have several bands at 1375, 1315, 1155, 1075, 1040, and 895 cm^−1^ characteristic for *β*-anomeric glucose units and, therefore, *β*-glucans [65,66,67,68,69].

### 3.6. NMR Spectra

The assignment of the proton and carbon signals of the main sugar units of the fractions **F1**, **F2**, and **F3** is summarized in Table 6. The assignment was made using semi-empirical calculations of Casper software (Future Systems Solutions, Inc., USA) [88] and using the literature [47,48,89].

**Figure 6:**
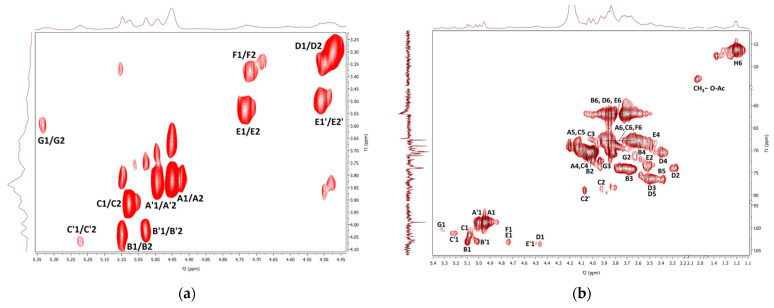
^1^H, ^1^H COSY (**a**) and ^1^H, ^13^C HMQC (**b**) NMR spectra of the fraction **F1**.

The COSY NMR spectrum of the fraction **F1** (Figure 6a) showed several H1α/H2 cross peaks assigned to, 1,6-linked (**A**, **A’**) and 1,2,6-linked α-d-galactosyl (**C**, **C’**), terminal *β*-d-mannosyl (**B**, **B’**), and 1,4-linked α-d-glucosyl (**G**) units. These units probably come from two polysaccharides, i.e., branched *O*-2-*β*-d-mannosyl-(1→6)-α-d-galactan, and, in smaller amounts, amylose-like (1→4)-α-d-glucan. In contrast to mannogalactans previously described for mushrooms of genus *Pleurotus* [36,90,91], this mannogalactan obtained from *G. resinaceum* was not methylated at the *O*-3 position of the backbone galactosyl units because no signals of OCH_3_ groups were found. Moreover, several H1*β*/H2 cross peaks were assigned to terminal *β*-d-glucosyl (**D**), 1,6-linked *β*-d-glucosyl (**D’**), 1,3-linked *β*-d-glucosyl (**E**), 1,3,6-linked *β*-d-glucosyl (**E’**), and 1,4-linked *β*-d-glucosyl (**F**, **F’**) units; all of them are probably parts of a highly branched (1→3)(1→4)(1→6)-*β*-d-glucan. The HMQC spectrum (Figure 6b) shows the signals that indicate the presence of the CHOH, CHOR, CH_2_OH, and CH_2_OR’ residues of the above polysaccharides. The HMQC signal of CH_3_ at 1.20 ppm/16.2 ppm and COSY cross peak at 1.20 ppm/4.13 ppm arose from the α-fucosyl units (**H**). The HMQC signal at 2.02 ppm/22.9 ppm indicated a small amount of *O*-acetyl groups. Therefore, according to the relative intensities of the resonance signals and previous analyses mentioned above, **F1** contains branched mannogalactan as the main product, lower content of branched *β*-glucan, and a negligible amount of α-glucan. Water-soluble heterogalactan has been previously isolated from the fruiting bodies of *Ganoderma atrum* [52]. As in the case of the current study, this polysaccharide had a backbone of 1,6-linked α-d-galactosyl units, but in this case, terminal α-l-fucosyl and α-d-mannosyl units and oligosaccharide fragments can act as side chains attached to the *O*-6 position of some of the backbone units.

**Figure 7:**
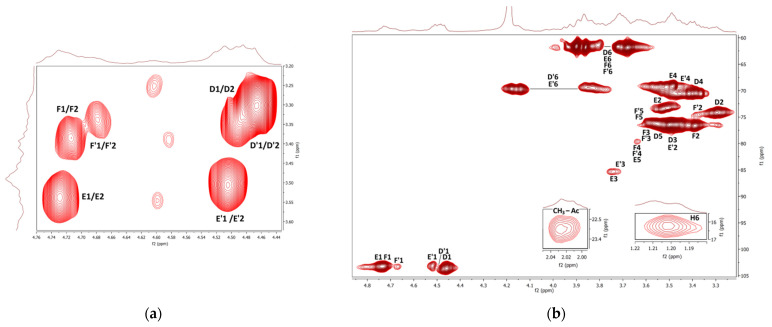
^1^H, ^1^H COSY (**a**) and ^1^H, ^13^C HMQC (**b**) NMR spectra of the fraction **F2**.

The COSY and HMQC NMR spectra of the fraction **F2** are shown in Figure 7a,b. As in the case of **F1**, branched (1→3)(1→6)-*β*-d-glucan is represented here by 1,3-linked, 1,4-linked, 1,6-linked, 1,3,6-linked, and terminal *β*-d-glucosyl units (**D–F**). Furthermore, the presence of mannogalactan was proved by the weak signals of 1,6-linked and 1,2,6-linked *α*-d-galactosyl and terminal *β*-d-mannosyl units (**A–C**). A weak HMQC signal at 2.03 ppm/22.96 ppm was assigned to CH_3_ of *O*-acetyl groups. In addition, 1,4-linked *α*-d-glucosyl (**G**) and *α*-l-fucosyl (**H**) units were also detected. In general, branched β-glucan predominated in this fraction, while *α*-glucan and mannogalactan were present to a lesser extent. Water-soluble highly branched *β*-glucans of similar structure have been previously described for some *Ganoderma species* [18]; these polysaccharides were isolated from fruiting bodies, and crowing culture of mycelium [92] and spores [93,94]. For example, water-soluble *β*-d-glucan was previously isolated from basidiocarps of *Ganoderma resinaceum* [95]. It was a highly branched polysaccharide that contained 1,3-linked *β*-d-glucosyl units in the backbone, partially substituted mainly by the side chains of 4-*O*-substituted *β*-d-glucosyl units at the *O*-6 on average for every two residues of the main chain.

**Figure 8:**
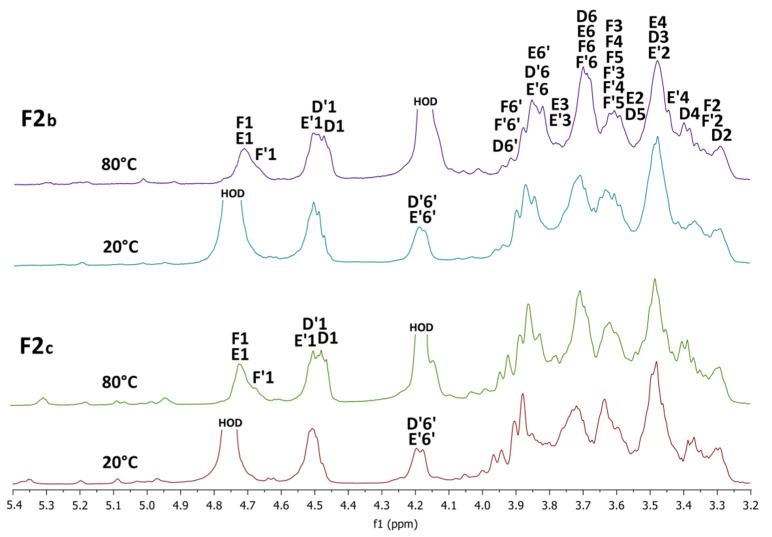
^1^H NMR spectra of the sub-fractions **F2b** and **F2c** measured in D_2_O at 20 °C and 80 °C.

The ^1^H NMR spectra of the **F2b** and **F2c** sub-fractions were measured in D_2_O at 20 °C and 80 °C and are shown in Figure 8. For both fractions, when measured at 20 °C, an intense HOD signal overlapped the H1β resonances, and when measured at 80 °C, this intense signal shifted upfield and already overlapped the H6 resonances. The spectra of both sub-fractions are very similar to the spectrum of the initial fraction **F2**, and only minor differences were observed between them. Within the fractions, there is certainly a different ratio between the signals of individual glucosyl residues. For example, the signals of terminal *β*-d-glucosyl units **D** were more pronounced in the case of sub-fraction **F2c**, so this product should have a higher degree of branching.

**Figure 9:**
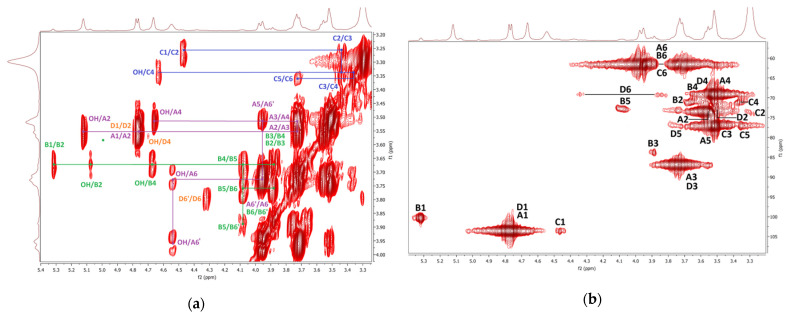
^1^H, ^1^H COSY (**a**) and ^1^H, ^13^C HMQC (**b**) NMR spectra of the fraction **F3**.

The COSY and HMQC NMR spectra of the fraction **F3** are represented in Figure 9. The spectra were measured in Me_2_SO-*d*_6_, so there is no proton exchange between the hydroxylic groups and the solvent, and the COSY spectra reveal both CH and OH proton signals (Figure 9a). The assignment of the proton and carbon signals of the main sugar units is shown in Table 6. The H1/H2 and OH/CH cross peaks with different intensities found in the COSY spectrum were assigned to the three main glucosyl units. The signals of 1,3-linked *β*-d-glucosyl units (**A**) were the most pronounced; the less intense signals were assigned to the 1,3-linked α-d-glucosyl (**B**), terminal *β*-glucosyl (**C**), and 1,3,6-linked *β*-d-glucosyl (**D**) residues. The signals of units **A** were also the most intense in the HMQC spectrum (Figure 9b). These units are evidently inter-connected into the (1→3)-*β*-d-glucan backbone that can be slightly branched at *O*-6 of the units **D** (branching points) by the terminal *β*-d-glucosyl units **C**. Slightly branched (1→3)(1→6)-*β*-d-glucan was isolated by alkaline extraction from the fruiting bodies of *Ganoderma japonicum* [96]. In this polysaccharide, only every 30th unit of the backbone was a branching point carrying a terminal *β*-d-glucosyl attached at the *O*-6 position. In contrast, the units **B** are rather linked to each other, yielding the (1→3)-*α*-d-glucan, which is less pronounced in **F3**. Pure *α*-d-glucan of a similar structure has been previously isolated from *Ganoderma lucidum* by alkaline extraction [89]. The alkaline extract from mycelium of *Ganoderma tsugae* contained the mixture of (1→3)-α-d-glucan and mannoxylan [97].

## 4. Conclusions

In this work, polysaccharide fractions of *G. resinaceum* extracted from dried fruiting bodies were used for a deeper investigation. Four polysaccharidic fractions, including the insoluble part, were obtained from the basidiocarp by successive extractions with cold water, hot water, and alkaline solution. The cold water extract was defined as a mixture of three polysaccharides: branched *O*-2-*β*-d-mannosyl-(1→6)-α-d-galactan, branched (1→3)(1→6)-*β*-d-glucan, and branched (1→6)(1→2)-*β*-d-galactan. The hot water extract consisted mainly of branched (1→3)(1→6)-*β*-d-glucan and linear (1→4)-α-d-glucan, but also contained a small amount of (1→2)(1→6)-*β*-d-galactan. Two sub-fractions of this extract were obtained by preparative chromatography; both contained branched (1→3)(1→6)-*β*-d-glucans, but differed in the degree of branching and in the presence of uronic acids. The alkaline extract consisted mainly of (1→3)-*β*-d-glucan slightly branched at *O*-6 by terminal *β*-glucose, and a small amount of (1→3)-α-d-glucan was also found in this fraction. Therefore, the *β*-d-glucans obtained by alkaline extraction were less branched than the water-soluble *β*-d-glucans. The solid residues after all of the extractions were defined as the complex of two main cell wall polysaccharides, i.e., chitin and *β*-d-glucan. The high content of d-glucans of various structures with potential immunomodulatory activity in basidiocarps of *Ganoderma resinaceum*, described in this study, makes this species promising for cultivation in order to obtain these polysaccharides, as well as other potentially biologically active substances for the preparation of functional food products or nutritional supplements that can support health and prevent the development of various diseases.

## Figures and Tables

**Table 1:** Yields and composition of the fractions obtained.

Fraction	Extraction Medium	Yield (% *w/w*)	Composition
**F1**	Cold water	0.37	Polysaccharides, proteins
**F2**	Hot water	2.33	Polysaccharides
**F3**	1 mol L^−1^ NaOH	10.57	Polysaccharides
**F4**	Insoluble residues	15.59	Polysaccharides
**Total**	All fractions	28.86	Polysaccharides, proteins

**Table 2:** Amounts of organic elements (% *w/w*) in the fractions obtained from basidiocarps of *G. resinaceum*.

Fraction	% N	% C	% H	% S
**F1**	2.44	39.47	6.25	0.13
**F2**	0.70	39.43	6.92	0.04
**F3**	0.21	39.53	7.13	0.21
**F4**	2.08	39.47	6.44	0.07

**Table 3:** Molar ratio (%) of monosaccharides in the fractions obtained from basidiocarps of *G. resinaceum*.

Fraction	Fuc	Man	Glc	Gal	Xyl
**F1**	6.7	26.5	35.6	26.9	4.3
**F2**	3.5	4.2	86.5	1.9	3.9
**F3**	2.0	2.7	92.2	0.0	3.1
**F4**	3.6	0.0	92.4	0.0	4.0

**Table 4:** Sugar linkage analysis of **F2**.

Sugar Derivative	Ratio (mol %)	Mode of Linkage
2,3,4,6-Me_4_-Man	Traces	Man*p*-(1→
2,3,4,6-Me_4_-Glc	22.1	Glc*p*-(1→
2,4-Me_2_-Fuc	1.1	→3)-Fuc*p*-(1→
2,3,4,6-Me_4_-Gal	0.5	Gal*p*-(1→
2,3-Me_2_-Xyl	0.2	→4)-Xyl-(1→
2,4,6-Me_3_-Glc	26.8	→3)-Glc*p*-(1→
2,3,4-Me_3_-Glc	12.8	→6)-Glc*p*-(1→
2,3,6-Me_3_-Glc	17.6	→4)-Glc*p*-(1→
2,3,4-Me_3_-Gal	1.6	→6)-Gal*p*-(1→
2,6-Me_2_-Man	0.4	→3,4)-Man*p*1→
2,4-Me_2_-Glc	14.2	→3,6)-Glc*p*-(1→
2,3-Me_2_-Man	1.9	→4,6)-Man*p*-(1→
3,4-Me_2_-Man	0.6	→2,6)-Man*p*-(1→

**Table 5:** Preparative chromatography of **F2**.

Fraction/Sub-Fraction		Weight (mg)	Yield (% *w/w*)
**F2**	Enter	66.67	
**F2a**	Minor	0.13	0.19
**F2b**	Major	32.54	48.81
**F2c**	Major	11.73	17.59

**Table 6:** The assignment of proton and carbon signals (ppm) of the main sugar units for the fractions **F1**, **F2**, and **F3** [30,66,67,73].

Fraction	Unit		H1/C1	H2/C2	H3/C3	H4/C4	H5/C5	H6/C6	O2H	O4H	O6H
**F1, F2**	**A**	→6)-*α*-Gal*p*-(1→	4.95	3.82	3.85	4.02	4.14	3.68; 3.85			
			98.8	68.9	70.3	70.4	69.5	67.3			
	**A’**	→6)-*α*-Gal*p*-(1→	5.00	3.83	3.85	4.02	4.14	3.68; 3.85			
			98.8	68.9	70.3	70.4	69.5	67.3			
	**B**	*β*-Man*p*-(1→2	5.10	4.04	3.68	3.60	3.33	3.70; 3.87			
			102.9	70.5	74.2	68.1	76.5	61.9			
	**B’**	*β*-Man*p*-(1→2	5.02	4.03	3.68	3.60	3.33	3.68; 3.85			
			102.9	70.5	74.2	68.1	76.5	61.9			
	**C**	→2,6)-*α*-Gal*p*-(1→	5.08	3.92	4.00	4.02	4.14	3.68; 3.85			
			101.6	78.48	67.7	70.4	69.5	67.3			
	**C’**	→2,6)-*α*-Gal*p*-(1→	5.22	4.06	3.99	4.02	4.14	3.68; 3.85			
			101.2	78.90	67.8	70.4	69.5	67.3			
	**D**	*β*-Glc*p*-(1→	4.47	3.30	3.48	3.38	3.61	3.78; 3.94			
			103.6	74.1	76.7	70.6	76.2	61.8			
	**D’**	→6)-*β*-Glc*p*-(1→	4.49	3.34	3.48	3.37	3.51	3.85; 4.15			
			103.6	74.3	76.7	70.6	76.5	69.3			
	**E**	→3)-*β*-Glc*p*-(1→	4.73	3.54	3.74	3.48	3.65	3.70; 3.87			
			103.2	73.4	85.3	69.2	79.6	61.8			
	**E’**	→3,6)-*β*-Glc*p*-(1→	4.52	3.50	3.70	3.41	3.72	3.85; 4.15			
			103.1	76.5	85.3	70.6	74.2	69.3			
	**F**	→4)-*β*-Glc*p*-(1→	4.71	3.39	3.63	3.63	3.60	3.78; 3.94			
			103.2	76.6	76.5	79.7	76.2	61.8			
	**F’**	→4)-*β*-Glc*p*-(1→	4.68	3.37	3.62	3.63	3.60	3.78; 3.94			
			103.3	74.5	76.5	79.7	76.2	61.8			
	**G**	→4)-*α*-Glc*p*-(1→	5.34	3.60	3.92	3.53	3.80	3.87			
			100.3	71.5	73.3	78.8	72.6	60.7			
	**H**	→3)-*α*-Fuc*p*-(1→					4.12	1.20			
								16.2			
**F3**	**A**	→3)-*β*-Glc*p*-(1→	4.76	3.56	3.73	3.48	3.51	3.73; 3.96	5.12	4.66	4.54
			103.6	73.4	86.9	69.1	79.1	61.7			
	**B**	→3)-*β*-Glc*p*-(1→	5.31	3.67	3.88	3.67	4.07	3.76; 3.88	5.07	4.67	
			100.3	71.3	83.7	69.0	72.8	61.6			
	**C**	*β*-Glc*p*-(1→6	4.47	3.26	3.44	3.34	3.38	3.71; 3.96		4.63	
			103.6	73.9	76.9	71.2	77.2	61.7			
	**D**	→3,6)-*β*-Glc*p*-(1→	4.76	3.53	3.72	3.57	3.77	3.83; 4.32		4.70	
			103.6	73.7	86.9	69.1	76.8	69.3			
